# Associations Between DAT, CBSE, and USMLE Step 1 Exams Among Oral and Maxillofacial Surgery Residents

**DOI:** 10.1002/jdd.70175

**Published:** 2026-02-16

**Authors:** Obadiah J. Pirlo, Felix J. Amarista, Daniel E. Perez

**Affiliations:** ^1^ University of Texas Health Science Center San Antonio Texas USA; ^2^ Department of Oral and Maxillofacial Surgery University of Texas Health Science Center San Antonio Texas USA

**Keywords:** educational assessment, educational measurement, school admission criteria

## Abstract

**Purpose/Objectives:**

The Dental Admission Test (DAT), Comprehensive Basic Science Examination (CBSE), and United States Medical Licensing Examination (USMLE) Step 1 are used to assess readiness in dental and medical education. This study examined associations between DAT scores (composite and subsections), CBSE scores, and USMLE Step 1 performance, to explore how these exams relate to each other.

**Methods:**

A cross‐sectional study was conducted using self‐reported Qualtrics survey responses from oral & maxillofacial surgery residents distributed in 2023. Respondents from MD‐granting programs reported their DAT, CBSE, and USMLE Step 1 scores. CBSE scores were standardized using the National Board of Medical Examiners (NBME) conversion chart to account for changes in score reporting. Of 77 responses, 27 met inclusion criteria. Due to unknown survey distribution totals, a formal response rate could not be calculated. Correlation analyses were performed, with statistical significance set at *p* < 0.05.

**Results:**

Significant positive correlations were found between DAT Academic Average, Total Science, General Chemistry, and Biology scores and standardized CBSE scores (*r* = 0.40–0.60; 95% CI: 0.15–0.75; *p* < 0.05). No significant associations were observed between DAT or CBSE scores and USMLE Step 1 performance (*r* = −0.10–0.20; 95% CI: −0.35–0.40; *p* > 0.05).

**Conclusions:**

DAT composite and select subsection scores were associated with CBSE performance but not USMLE Step 1 outcomes. These results suggest that while the DAT may reflect early medical science readiness, it does not predict downstream Step 1 performance.

## 1 Introduction

The Dental Admission Test (DAT), Comprehensive Basic Science Examination (CBSE), and United States Medical Licensing Examination (USMLE) Step 1 are standardized assessments used by dental and medical education programs to evaluate foundational knowledge and critical thinking skills. The DAT assesses science knowledge (biology, general chemistry, organic chemistry sciences, and quantitative reasoning), and skills in perceptual ability and reading comprehension. Prior studies have shown that DAT scores can predict performance in predoctoral dental curricula and on the National Board Dental Examination (NBDE) Part I [1, 2].

NBDE Part I results have been shown to predict USMLE Step 1 performance, highlighting overlap between these exams [3]. The CBSE, developed by the National Board of Medical Examiners (NBME), is widely used by medical schools to assess readiness for the USMLE Step 1, a critical exam for licensure and residency selection [4].

Oral and maxillofacial surgery (OMS) residents in dual‐degree programs uniquely undergo all three examinations—DAT, CBSE, and USMLE Step 1—during their training [5, 6]. While correlations between CBSE and USMLE Step 1 performance have been demonstrated in traditional medical students [7, 8, 9, 10], limited research has examined whether earlier assessments like the DAT are predictive of later academic success in OMS pathways.

This study aimed to determine whether DAT composite and subsection scores are correlated with CBSE and USMLE Step 1 outcomes among OMS residents. By exploring these relationships, we seek to assess whether early academic indicators may serve as valid predictors of performance on subsequent high‐stakes medical licensing examinations. We hypothesize that higher DAT composite and subsection scores are strongly associated with CBSE and USMLE Step 1 performance among OMS residents.

## 2 Materials and Methods

### 2.1 Ethical Approval

This study was reviewed and approved by the Institutional Review Board (IRB) at the University of Texas Health Science Center at San Antonio (Protocol No. 20230554EX). All data were de‐identified, and no personal or demographic information was collected to ensure participant confidentiality.

[Correction added on February 21, 2026, after first online publication: The Ethical Approval section has been updated in this version.]

### 2.2 Study Design

This was a cross‐sectional study aimed at examining correlations among DAT, CBSE, and USMLE Step 1 scores among OMS residents. A survey was developed and distributed electronically via Qualtrics to program directors of all 44 accredited MD‐granting OMS residency programs in the United States in 2023. Program directors were asked to forward the survey to their current residents.

### 2.3 Participants and Inclusion Criteria

A total of 77 survey responses were received. Of these, 27 responses met the inclusion criteria and were included in the final analysis. To be included, participants had to report complete data for DAT Academic Average, Total Science, and all subsection scores (General Chemistry, Organic Chemistry, Biology, Quantitative Reasoning, Reading Comprehension, and Perceptual Ability), as well as standardized CBSE scores and USMLE Step 1 results in either numeric or pass/fail format. Responses with incomplete or missing data (*n* = 50) were excluded.

### 2.4 Data Processing and Standardization

CBSE scores were standardized using the NBME approximate score equivalency chart to account for changes in score reporting. USMLE Step 1 scores were reported in numeric format where available, or as pass/fail in alignment with the transition to pass/fail scoring implemented on January 26, 2022.

### 2.5 Statistical Analysis

Descriptive statistics were computed to summarize DAT, CBSE, and USMLE Step 1 scores. Pearson correlation analyses were performed to evaluate the strength and direction of relationships between DAT composite and subsection scores, CBSE performance, and numeric USMLE Step 1 results. Correlation coefficients (*r* values) were interpreted using conventional thresholds: Weak (<0.30), moderate (0.30–0.59), and strong (≥0.60). Confidence intervals were calculated for each correlation to assess statistical precision. All analyses were conducted using R Studio (Version 2024.12.0+467), and a two‐tailed *p*‐value of less than 0.05 was considered statistically significant.

## 3 Results

A total of 77 OMS residents responded to the survey, of which 27 (35%) met the inclusion criteria for complete data reporting. Of these, 8 (30%) residents provided numeric USMLE Step 1 scores, with a mean of 230 and a standard deviation of 21, while 19 (70%) reported only pass/fail outcomes—all of whom passed. Descriptive statistics for DAT composite scores, subsection scores, CBSE scores, and numeric USMLE Step 1 scores are presented in Table 1.

**TABLE 1:** Descriptive statistics of DAT subsections, CBSE scores, and USMLE Step 1 outcomes among oral and maxillofacial surgery (OMS) residents (*n* = 27). Values are presented as mean ± standard deviation (SD) for continuous variables and as *n* (%) for categorical variables. USMLE Step 1 numeric scores were reported for 8 participants, while 19 participants provided pass/fail outcomes only.

Characteristic	*n* = 27^1^
DAT Academic Average	23.15 ± 2.25
DAT Total Science	23.8 ± 2.9
DAT Organic Chemistry	24.7 ± 3.6
DAT General Chemistry	23.33 ± 2.75
DAT Biology	23.2 ± 3.6
DAT Reading Comprehension	23.04 ± 2.34
DAT Quantitative Reasoning	22.2 ± 3.4
DAT Perceptual Ability	21.78 ± 2.01
CBSE Score	220 ± 19
USMLE Step 1 Pass/Fail	
Pass	27 (100%)
Numeric USMLE Step 1 score	230 ± 21
Unknown	19
^1^Mean ± SD; *n* (%)	

Correlation analyses revealed statistically significant associations between CBSE scores and several components of the DAT. Specifically, CBSE performance demonstrated significant positive correlations with DAT Academic Average (*r* = 0.42, *p* = 0.03), Total Science (*r* = 0.53, *p* = 0.005), General Chemistry (*r* = 0.49, *p* = 0.01), and Biology (*r* = 0.60, *p* < 0.001).

No significant correlations were found between DAT scores or CBSE scores and numeric USMLE Step 1 performance. While the correlation between CBSE and USMLE Step 1 numeric scores was moderate (*r* = 0.66), it did not reach statistical significance (*p* > 0.05), which may be attributed to the limited sample size of available numeric scores.

Table 2 presents the Pearson correlation coefficients between all exam variables, with significant findings noted. Table 3 provides the corresponding *p*‐values. Figure 1 illustrates a matrix plot showing all pairwise relationships among the DAT, CBSE, and USMLE Step 1 scores. Figure 2 presents a color‐coded heatmap to visually emphasize significant correlations and enhance interpretability.

**TABLE 2:** Pearson correlation coefficients between DAT subsections, CBSE scores, and numeric USMLE Step 1 scores. *Significant correlations (p < 0.05) are marked with an asterisk* (*). USMLE Step 1 numeric scores were reported by 8 participants, limiting statistical power for correlations with this variable.

	DAT Academic Average	DAT Total Science	DAT Organic Chemistry	DAT General Chemistry	DAT Biology	DAT Reading Comprehension	DAT Quantitative Reasoning	DAT Perceptual Ability	CBSE Score	Numeric USMLE Step 1 Score
DAT Academic Average	1	0.87*	0.8*	0.77*	0.8*	0.43*	0.52*	0.33	0.42*	0.26
DAT Total Science	0.87*	1	0.88*	0.76*	0.89*	0.26	0.36	0.14	0.53*	0.41
DAT Organic Chemistry	0.8*	0.88*	1	0.51*	0.81*	0.2	0.29	0.01	0.38	0.48
DAT General Chemistry	0.77*	0.76*	0.51*	1	0.64*	0.24	0.38*	0.41*	0.49*	0.07
DAT Biology	0.8*	0.89*	0.81*	0.64*	1	0.39*	0.21	−0.15	0.6*	0.56
DAT Reading Comprehension	0.43*	0.26	0.2	0.24	0.39*	1	0.19	−0.03	−0.03	0.27
DAT Quantitative Reasoning	0.52*	0.36	0.29	0.38*	0.21	0.19	1	0.56*	0.16	0.29
DAT Perceptual Ability	0.33	0.14	0.01	0.41*	−0.15	−0.03	0.56*	1	0	−0.29
CBSE Score	0.42*	0.53*	0.38	0.49*	0.6*	−0.03	0.16	0	1	0.66
Numeric USMLE Step 1 Score	0.26	0.41	0.48	0.07	0.56	0.27	0.29	−0.29	0.66	1

**TABLE 3:** *p*‐values for relationships between DAT subsections, CBSE scores, and numeric USMLE Step 1 scores based on Pearson correlation analysis.

	DAT Academic Average	DAT Total Science	DAT Organic Chemistry	DAT General Chemistry	DAT Biology	DAT Reading Comprehension	DAT Quantitative Reasoning	DAT Perceptual Ability	CBSE Score	Numeric USMLE Step 1 Score
DAT Academic Average	n/a	<0.001	<0.001	<0.001	<0.001	0.025	0.005	0.091	0.030	0.5
DAT Total Science	<0.001	n/a	<0.001	<0.001	<0.001	0.2	0.062	0.5	0.005	0.3
DAT Organic Chemistry	<0.001	<0.001	n/a	0.00.7	<0.001	0.3	0.14	>0.9	0.054	0.2
DAT General Chemistry	<0.001	<0.001	0.007	n/a	<0.001	0.2	0.047	0.033	0.010	0.9
DAT Biology	<0.001	<0.001	<0.001	<0.001	n/a	0.042	0.3	0.4	<0.001	0.2
DAT Reading Comprehension	0.025	0.2	0.3	0.2	0.042	n/a	0.3	0.9	0.9	0.5
DAT Quantitative Reasoning	0.005	0.062	0.14	0.047	0.3	0.3	n/a	0.002	0.4	0.5
DAT Perceptual Ability	0.091	0.5	>0.9	0.033	0.4	0.9	0.002	n/a	>0.9	0.5
CBSE Score	0.030	0.005	0.054	0.010	<0.001	0.9	0.4	>0.9	n/a	0.077
Numeric USMLE Step 1 Score	0.5	0.3	0.2	0.9	0.2	0.5	0.5	0.5	0.077	n/a

**FIGURE 1:**
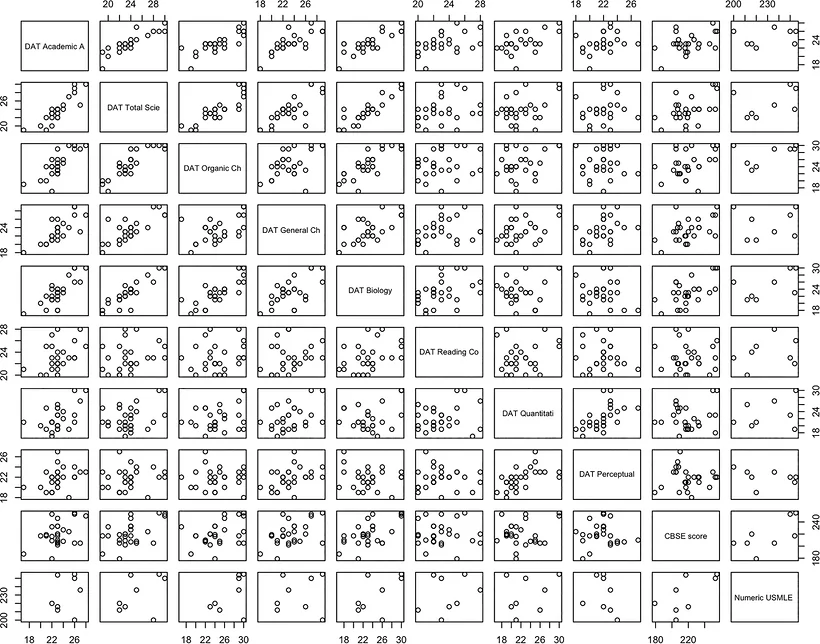
Pairwise scatterplot matrix of DAT composite scores, CBSE scores, and numeric USMLE Step 1 scores. Each panel represents the relationship between two variables, with data points plotted for individual participants. The diagonal panels show distributions for each variable, while off‐diagonal panels display scatterplots indicating the strength and direction of relationships. This visualization highlights where meaningful associations exist—and where they do not—providing a broad overview of the relationships among DAT subsection performance, CBSE outcomes, and USMLE Step 1 results in dual‐degree OMS residents.

**FIGURE 2:**
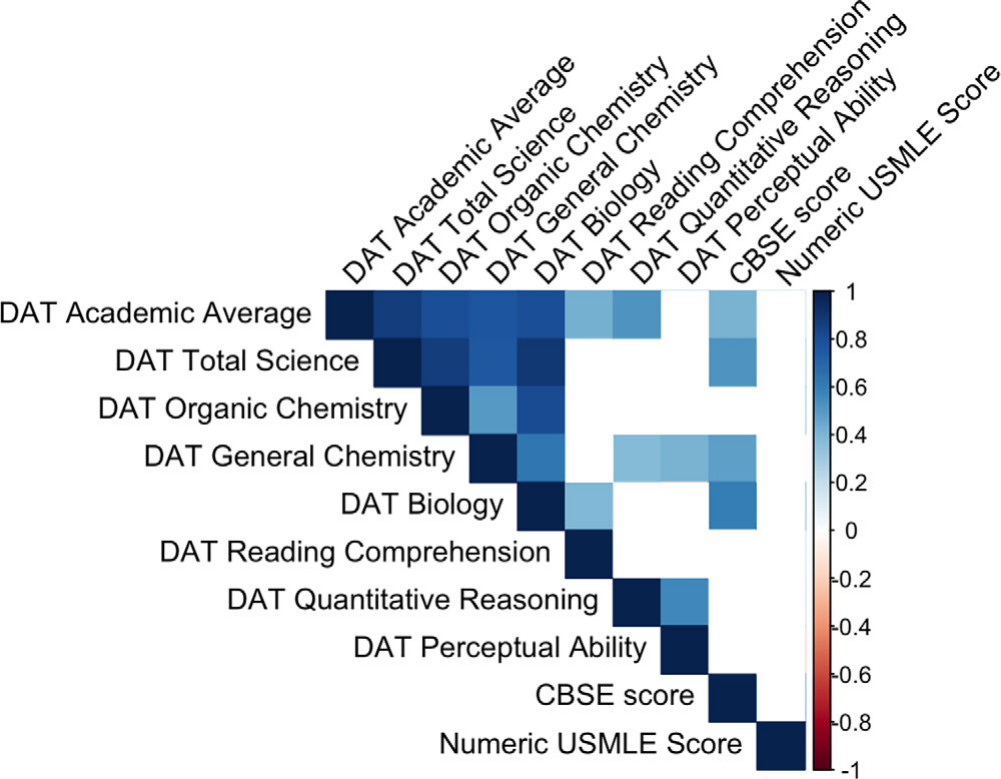
Significant correlations from Table 1 shown within color heatmap. The following were correlated with CBSE score: DAT Academic Average, DAT Total Science, DAT General Chemistry, and DAT Biology.

## 4 Discussion

This study investigated the relationships among DAT scores, CBSE performance, and USMLE Step 1 outcomes in OMS residents, a unique cohort that bridges both dental and medical education. The findings demonstrated that higher DAT composite and subsection scores—particularly in Biology and General Chemistry—were significantly associated with stronger CBSE performance. However, neither DAT nor CBSE scores were significantly associated with USMLE Step 1 outcomes.

The observed associations between DAT scores and CBSE performance reinforce prior research suggesting that the DAT serves as a reliable early predictor of success in dental education and related examinations such as the NBDE Part I [1, 2]. This study extends those findings by suggesting that among dual‐degree OMS residents, the DAT—especially science‐related subsections—may have utility in predicting performance on medical school‐based assessments like the CBSE. Biology and General Chemistry, foundational to both dental and medical basic science curricula, showed the strongest correlations, aligning with their central role in preclinical training.

In contrast, no significant correlations were found between DAT or CBSE scores and numeric USMLE Step 1 scores. While the CBSE has demonstrated predictive validity for Step 1 outcomes in traditional medical students [7, 8, 9], the lack of correlation in this study may reflect the non‐traditional trajectory of OMS residents, who enter medical curricula after dental school with variable timing and prior exposure. These differences may limit the generalizability of performance predictors derived from single‐discipline training models [5, 10].

Another consideration is the small sample size of residents reporting numeric USMLE Step 1 scores (*n* = 8), which reduces statistical power and may obscure true relationships. The recent transition of Step 1 to pass/fail reporting further limits its utility as a comparative metric, as program directors and institutions are now less able to differentiate performance on a fine‐grained scale [12]. In addition, non‐cognitive factors such as test‐taking skills, resilience, and time management likely play a greater role in Step 1 success than foundational science knowledge alone [11].

These findings suggest that while the DAT is a useful tool for predicting early academic performance in OMS residents—as measured by CBSE outcomes—it should not be relied upon to forecast USMLE Step 1 performance. Similarly, the CBSE's predictive power for Step 1 may not translate well to dual‐degree trainees due to their non‐linear progression through medical education. Therefore, admissions committees and program directors should adopt a holistic approach, weighing DAT and CBSE scores alongside clinical performance, academic history, adaptability, and other non‐cognitive attributes. Complementary measures such as structured clinical evaluations, faculty assessments of surgical aptitude, professionalism, and letters of recommendation may provide a more nuanced view of readiness, helping programs balance academic performance with clinical potential.

This study contributes to a limited body of literature examining dual‐degree OMS residents, but it is not without limitations. First, the reliance on self‐reported data introduces potential for reporting bias. Second, the small number of complete datasets, especially for numeric USMLE Step 1 scores, limits the generalizability of the findings. Third, the cross‐sectional design precludes causal inferences and may not fully capture temporal relationships between exams. Fourth, because the survey was distributed indirectly through program directors and participation was voluntary, the study is subject to potential selection bias. It is possible that residents who were more academically engaged or confident in their test performance were more likely to respond, while others opted not to participate. This self‐selection may have introduced systematic differences between respondents and nonrespondents, potentially influencing the strength or direction of observed associations. In addition, the total number of OMS residents eligible to receive the survey could not be accurately determined, as the national population of dual‐degree OMS residents in inherently small and dynamic, limiting the feasibility of large‐sample studies. National resident rosters fluctuate yearly due to unfilled positions, varying program service start times, attrition, and temporary leaves during dual‐degree training, all of which preclude calculation of a meaningful response rate [13, 14]. Together, these factors limit the generalizability of the results, and the findings should be interpreted as preliminary.

Future research should aim to expand the sample size through multi‐institutional collaboration and longitudinal tracking. In addition, investigations into non‐cognitive predictors of success, such as resolve, emotional intelligence, and learning strategies, could offer valuable insights. As standardized testing continues to evolve, particularly with the ongoing de‐emphasis of numeric Step 1 scores, research must adapt to identify new markers of academic and clinical readiness in OMS and other dual‐degree pathways.

## 5 Conclusion

In summary, this study found that DAT Academic Average, Total Science, Biology, and General Chemistry scores significantly correlate with CBSE performance in OMS residents, supporting their utility as early indicators of academic strength. However, neither DAT nor CBSE scores predict USMLE Step 1 performance in this unique cohort. These findings emphasize the need for broader evaluation metrics in OMS training, incorporating not only exam performance but also clinical evaluations, professionalism, and interpersonal skills, to more accurately capture dual‐degree readiness. Future research should explore multi‐institutional datasets and longitudinal tracking to increase sample size.

## Disclosure

The authors have no financial, economic, or professional interests that influenced the design, execution, or presentation of this study.
